# Comparing Hypofractionated With Conventional Fractionated Radiotherapy After Breast-Conserving Surgery for Early Breast Cancer: A Meta-Analysis of Randomized Controlled Trials

**DOI:** 10.3389/fonc.2021.753209

**Published:** 2021-10-01

**Authors:** Lihu Gu, Wei Dai, Rongrong Fu, Hongfeng Lu, Jingyi Shen, Yetan Shi, Mengting Zhang, Ke Jiang, Feng Wu

**Affiliations:** ^1^ Department of General Surgery, HwaMei Hospital, University of Chinese Academy of Sciences, Ningbo, China; ^2^ Ningbo Institute of Life and Health Industry, University of Chinese Academy of Sciences, Ningbo, China; ^3^ The Second Clinical Medical College, Zhejiang Chinese Medical University, Hangzhou, China; ^4^ The First Clinical Medical College, Zhejiang Chinese Medical University, Hangzhou, China; ^5^ Department of Breast Surgery, HwaMei Hospital, University of Chinese Academy of Sciences, Ningbo, China

**Keywords:** breast cancer, hypofractionated radiotherapy, conventional fractionated radiotherapy, breast-conserving surgery, meta-analysis

## Abstract

**Background:**

The purpose of this meta-analysis was to compare the safety and efficacy between hypofractionated and conventional fractionation radiotherapy in patients with early-stage breast cancer after breast-conserving surgery.

**Methods:**

We conducted a comprehensive search of PubMed, Embase, Web of Science, and the Cochrane Library to identify relevant randomized controlled trials (RCTs) published before February 2021. At the same time, the hazard ratio (HR), risk ratio (RR), and 95% confidence interval (CI) were calculated to evaluate local recurrence (LR), relapse-free survival (RFS), overall survival (OS), adverse events, and cosmetic outcomes.

**Results:**

A total of 14 articles were included in this meta-analysis. Four thousand eight hundred and sixty-nine patients were randomly assigned to the control group to receive conventional radiotherapy (CFRT); 6,072 patients were randomly assigned to the experimental group and treated with hypofractionated radiotherapy (HFRT). The results showed that there was no statistical difference between HFRT and CFRT in LR (HR = 0.99, 95%CI = 0.97–1.02, *p* = 0.476), RFS (HR = 0.99, 95%CI = 0.97–1.02, *p* = 0.485), OS (HR = 1.00, 95%CI = 0.97–1.03, *p* = 0.879), and cosmetic outcomes (RR = 1.03, 95%CI = 0.95–1.12, *p* = 0.53). In addition, HFRT showed fewer severe adverse reactions such as acute skin toxicity, induration, breast atrophy, and pain.

**Conclusion:**

Our results suggest that there is no statistical difference between HFRT and CFRT in terms of LR, RFS, OS, and cosmetic outcomes. HFRT reduces the risk of developing toxicity reactions compared to CFRT. HFRT may be a better option for patients with early breast cancer after breast-conserving surgery.

## Introduction

According to the data released by the International Agency for Research on Cancer (IARC) of the World Health Organization in 2020, increased number of cases have made breast cancer become the world’s leading cancer by overtaking lung cancer (1). With the development of medical technology, the overall prognosis of breast cancer is good. In most countries and regions, the 5-year net survival rate is over 85%, and the trend is still rising (2). Patients in the early stage of breast cancer can choose between lumpectomy and mastectomy (3). Studies have shown that whole-breast radiotherapy following breast-conserving surgery is comparable to mastectomy in terms of overall survival (OS) in early breast cancer (4). Meanwhile, total mastectomy results in a larger wound area. Also, in addition to leading to a few unfavorable complications, total mastectomy also has negative impacts on patients’ social–emotional function and body image, which also affects patients’ self-esteem (5). Breast-conserving surgery, however, can improve the quality of life in a behavioral aspect compared to total mastectomy (6).

For patients in the early stage of breast cancer, radiotherapy after breast-conserving surgery is quite essential. The meta-analysis from the Early Breast Cancer Trialists’ Collaborative Group (EBCTCG) showed that the tumor recurrence and mortality rates were significantly reduced after breast-conserving surgery in patients who received whole-breast radiotherapy compared to those who did not (7). Based on this dominantly advantageous curative effect, it is time to consider the cosmetic outcomes, patients’ mental health, and the economic impact after surgery (8). The current conventional fractionated radiotherapy (CFRT) is 50 Gy fractionation divided into 25 fractions of 2 Gy over 5 weeks, once a day (9). However, this daily treatment takes five or more weeks, causing inconvenience to patients in terms of life and work, not to mention the negative impact on their social–emotional function (10). Hence, scholars have attempted to shorten the course of treatment. The aim of hypofractionated radiotherapy (HFRT) is to shorten the overall duration of treatment for patients by increasing the single dose of radiation, thereby providing greater convenience while bringing greater cost effectiveness and less resource waste to the entire healthcare system (11). The commonly used hypofractionated scheme is 43.5 Gy in 15 fractions over 15 days or 42.56 Gy in 16 fractions over 16 days (12). Due to the increase of the single dose and the decrease of the total dose, a great concern for HFRT has been whether to increase the toxicity and reduce the tumor control rate or not (13).

Four classical multicenter randomized controlled trials (RCTs) (14–17) have compared HFRT and CFRT and indicated equal local control, overall survival, and cosmetic outcomes, paving a promising future for the clinical application of HFRT (18). Although HFRT is theoretically promising in terms of shortening the course of treatment, it has no wide practical applications. This is probably due to the lingering concerns about the effectiveness and safety of HFRT (19). Thus, HFRT needs to be further evaluated in those aspects in order to improve our understanding and confidence in its clinical use. Herein, we conducted this meta-analysis to compare the treatment results of HFRT and CFRT in patients with early breast cancer after breast-conserving surgery in order to evaluate the safety and efficacy of HFRT.

## Methods

### Search Strategy

This meta-analysis was conducted in accordance with the Preferred Reporting Items for Systematic Reviews and Meta-Analyses (PRISMA) (20). The objective was to directly compare the safety and effectiveness through RCTs between HFRT and CFRT among early breast cancer patients who had previous breast-conserving surgery.

Two investigators independently performed the systematic and comprehensive search of databases such as PubMed, Embase, Web of Science, and the Cochrane Library for articles published before February 2021 using the following keywords, individually or in combination: (“breast cancer” OR “breast neoplasms” OR “breast tumor”) AND (“radiotherapy” OR “conventional fractionation” OR “hypofractionated” OR “hypofractionation radiotherapy dosage” OR “dose fractionation”). Moreover, the bibliographies of relevant publications were manually searched for additional articles.

### Selection Criteria

The included studies met the following criteria: 1) patients were histologically diagnosed with breast cancer; 2) patients have undergone breast-conserving surgery; 3) patients were treated with postoperative HFRT or CFRT; 4) HFRT should not be less than 2.3 Gy per fraction and the total dose should be more than 28 Gy; CFRT should be 1.8–2.0 Gy per fraction and the total dose should not be less than 45 Gy; and 5) relevant outcomes included but not limited to survival outcomes, tumor local control, toxicity, and cosmetic outcomes.

The exclusion criteria included the following: 1) the diagnosis was metastatic breast cancer; 2) data source was duplicated; 3) language of the article is non-English; 4) non-RCTs, including conference abstracts, observational research, review articles, and cases; 5) the efficacies of HFRT and CFRT were not directly compared; and 6) the data of the research are not feasible to be extracted.

### Data Extraction

Two investigators examined the data independently. The following pieces of information were extracted for each qualifying study: 1) study characteristics, including author, publication year, and country; 2) patient demographics, such as the clinical stage of breast cancer, sample size, radiation dose, median follow-up time, age range, systemic therapy, and time period of the clinical trials; 3) comparison of the clinical outcomes of HFRT and CFRT, including survival outcomes, tumor local control, toxicity, and cosmetic outcomes.

### Quality Assessment

To evaluate the RCTs, we used the Cochrane Collaboration’s tool. Two investigators independently used “low risk,” “high risk,” or “unclear risk” to assess the risk of bias through six aspects: sequence generation, allocation concealment, blinding, incomplete outcome data, selective outcome reporting, and free of other bias. Disagreements between the investigators were solved by consensus; if needed, a third investigator participated in the resolution process.

### Statistical Analysis

Meta-analysis was conducted by Review Manager, version 5.3, and Stata software, v.12.0. For categorical variables, such as adverse events and cosmetic outcomes, the risk ratio (RR) and 95% confidence interval (CI) were calculated for analysis. Meanwhile, survival data were analyzed by calculating the hazard ratio (HR) and 95% CI. *I* ^2^ was used to evaluate heterogeneity among the studies. A random effects model or a fixed effects model was applied to the analysis when the *I* ^2^ was greater than 50%. When more than 10 studies were included in the outcome indicators, the Begg’s and Egger’s tests are good tools to investigate publication bias. In addition, the stability and reliability of the results were evaluated using sensitivity analysis; thus, the sources of heterogeneity were further explored. *P* < 0.05 indicates statistical significance.

## Results

### Description of the Studies

**Figure 1:**
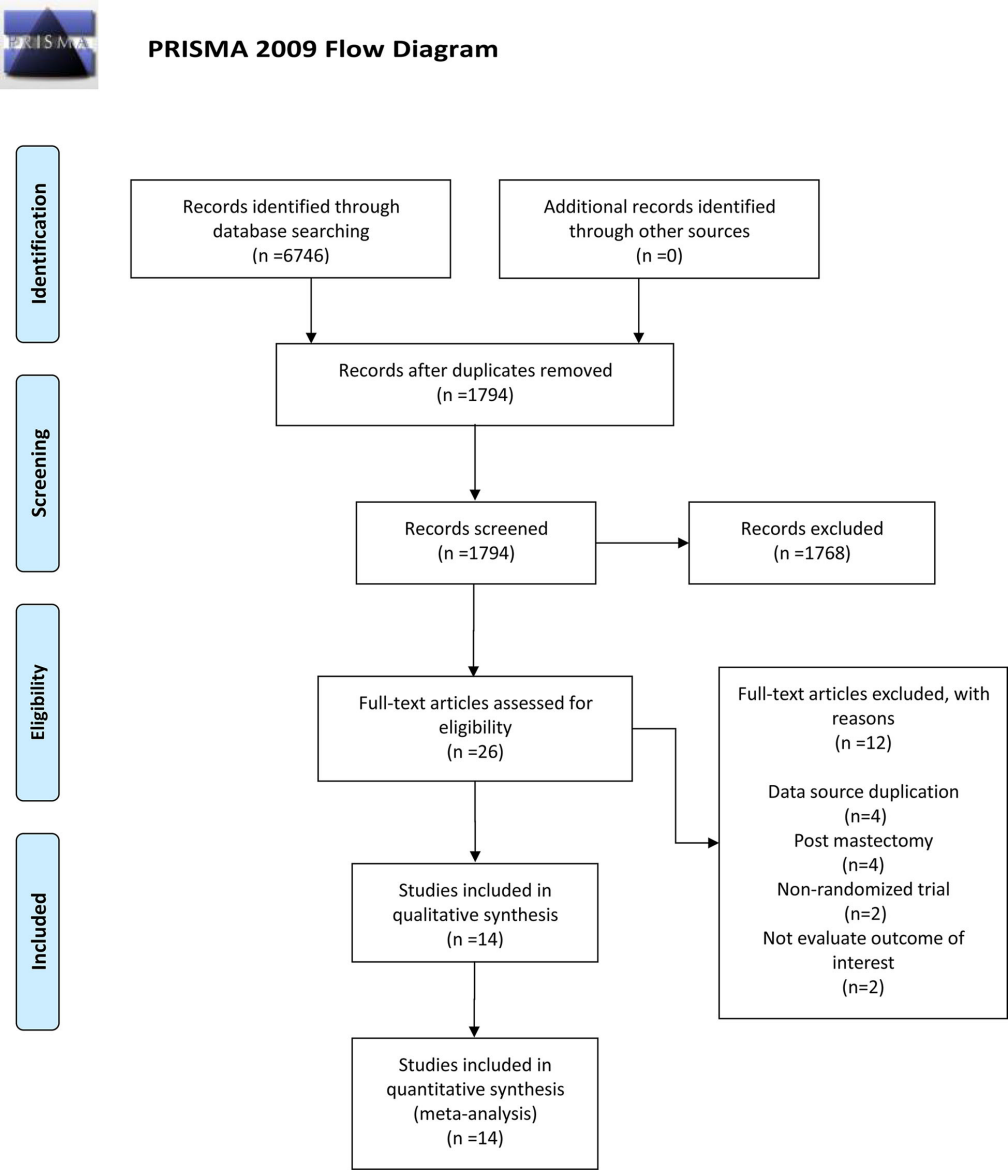
Flowchart of article selection for meta-analysis.

On the basis of our search strategy, a total of 6,746 records were identified from the four databases. After deleting duplicates, we screened the titles and abstracts of 1,794 records, then excluded 1,768 records and finally selected 26 to read through. We excluded 12 studies for the following reasons: four reported duplicate data sources; two were non-randomized controlled trials; two did not include relevant results; and the patients in four studies underwent mastectomy. Finally, we identified 14 studies that met the inclusion criteria. This meta-analysis is based on the quantitative and qualitative synthesis of the included 14 studies (14, 15, 21–32). A flowchart outlines the process in **Figure 1**.

More than 10,000 breast cancer cases were enrolled in the 14 included RCTs between 2004 and 2020. The characteristics of the studies are summarized in **Table 1** and **Supplementary Table S1**. Among these studies, four were conducted in the UK, three were in China, and seven were from other countries. All cases involved adult patients with non-metastatic breast cancer, mostly at T1-2N0-1, and all of the patients had undergone breast-conserving surgery. Of the total patients, 4,869 were randomly assigned to the control group and received CFRT with a radiotherapy dose of 1.8–2.0 Gy per fraction and a total dose of not less than 45 Gy (with or without a boost); 6,072 were randomly assigned to the experimental group and received HFRT treatment. The HFRT dose was more than 2.3 Gy per fraction, with a total dose of more than 28 Gy (with or without a boost). The shortest median follow-up time was 6 weeks and the longest was 16.9 years. The primary endpoints of the studies generally included survival rate, local control, toxicity, and cosmetic outcome.

**Table 1:** Characteristics of the included clinical trials in the meta-analysis.

First author	Year	Country	Clinical stage	Sample size	Intervention		Median follow-up
					Experiment	Control	
Wang (31)	2020	China	T1-2N0-3	734	43.5 Gy/15 fractions/3 weeks + tumor bed boost 8.7 Gy/3 daily fractions	50 Gy/25 fractions/5 weeks + tumor bed boost 10 Gy/5 fractions/1 week	73.5 months
Offersen (32)	2020	Denmark	DCIS or T1-2N0-1	1,882	40 Gy/15 fractions	50 Gy/25 fractions	7.3 years
Schmeel (27)	2020	Germany	T1-2 or DCIS	140	40.05 Gy/15 fractions	50 Gy/25 fractions	6 weeks
Shaitelman (26)	2018	USA	Tis-T2N0-1	286	42.56 Gy/16 fractions + 10–12.5 Gy/4–5 fractions	50 Gy/25 fractions + 10–14 Gy/5–7 fractions	4.1 years
Zhao (25)	2017	China	T1-2N0-1	107	42.56 Gy/16 fractions + tumor bed boost 7.98 Gy/3 fractions	50 Gy/25 fractions + tumor bed boost 10 Gy/5 fractions	122 months
De Felice (24)	2017	Italy	T1-2N0-1	120	42.5 Gy/16 fractions + 10 Gy/5fractions	50 Gy/25fractions + 10 Gy/5 fractions	16 months
Hashemi (23)	2016	Iran	T1-3N0	52	42.5 Gy/16 fractions	50 Gy/25 fractions	52.4 months
Hou (22)	2015	China	T1-2N0-1	80	43.2 Gy/18 fractions + boost to tumor bed 50.4 Gy/18 fractions	45 Gy/25 fractions + boost to tumor bed 59 Gy/7 fractions	27 months
Fragkandrea (30)	2013	UK	T1-2N0	61	43.2 Gy/16 fractions/22 days + boost 10 Gy/5 fractions/1 week	50 Gy/25 fractions/5 weeks + boost 10 Gy/5 fractions/1 week	NA
Haviland (28)	2013	UK	T1–3aN0–1	2,236	41.6 Gy/13 fractions or 39 Gy/13 fractions/5 weeks	50 Gy/25 fractions	9.3 years
				2,215	40 Gy/15 fractions/3 weeks		9.9 years
Spooner (21)	2012	UK	Stages I–II	707	40 Gy/15 fractions	50 Gy/25 fractions	16.9 years
Whelan (15)	2010	Canada	T1-2N0	1,234	42.5 Gy/16 fractions	50 Gy/25 fractions	12 years
Owen (14)	2006	UK	T1-3N0-1	1,410	42.9 Gy/13 fractions 39 Gy/13 fractions	50 Gy/25 fractions	9.7 years
Taher (29)	2004	Egypt	T1-2N0	30	42.5 Gy/16fractions/22 days	50 Gy/25 fractions/5 weeks + boost 10 Gy/5 fractions/1 week to tumor bed	23 months

DCIS, ductal carcinoma in situ; NA, not available.

### Risk of Bias in the Included Studies

The quality of each study was appraised *via* the Cochrane Collaboration’s tool. Most studies have been evaluated as low risk in terms of sequence generation and allocation concealment. However, the blinding of these studies was evaluated as having high risk of bias because the blinding process cannot be implemented due to the nature of the intervention. One study had high risk resulting from incomplete outcome data, and one had unclear risk. When it comes to selective outcome reporting, one study had high risk and one study was unclear. Finally, all RCTs were unclear in terms of free of other bias. The assessment results are shown in detail in **Supplementary Table S2**.

### Outcomes of Meta-Analysis

**Figure 2:**
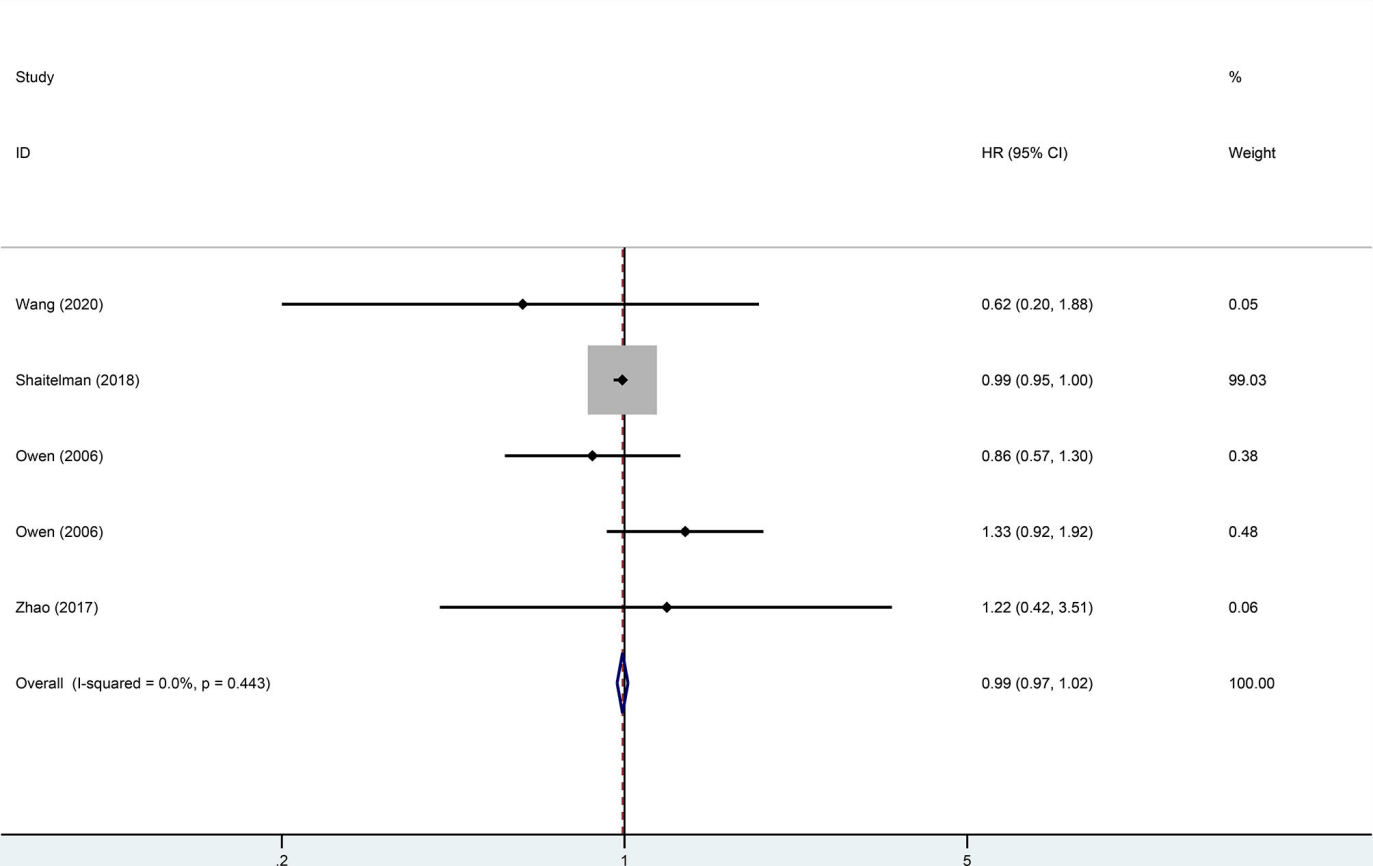
Forest plot of hypofractionated radiotherapy (HFRT) *vs*. conventional radiotherapy (CFRT) for local recurrence (*p* = 0.476).

**Figure 3:**
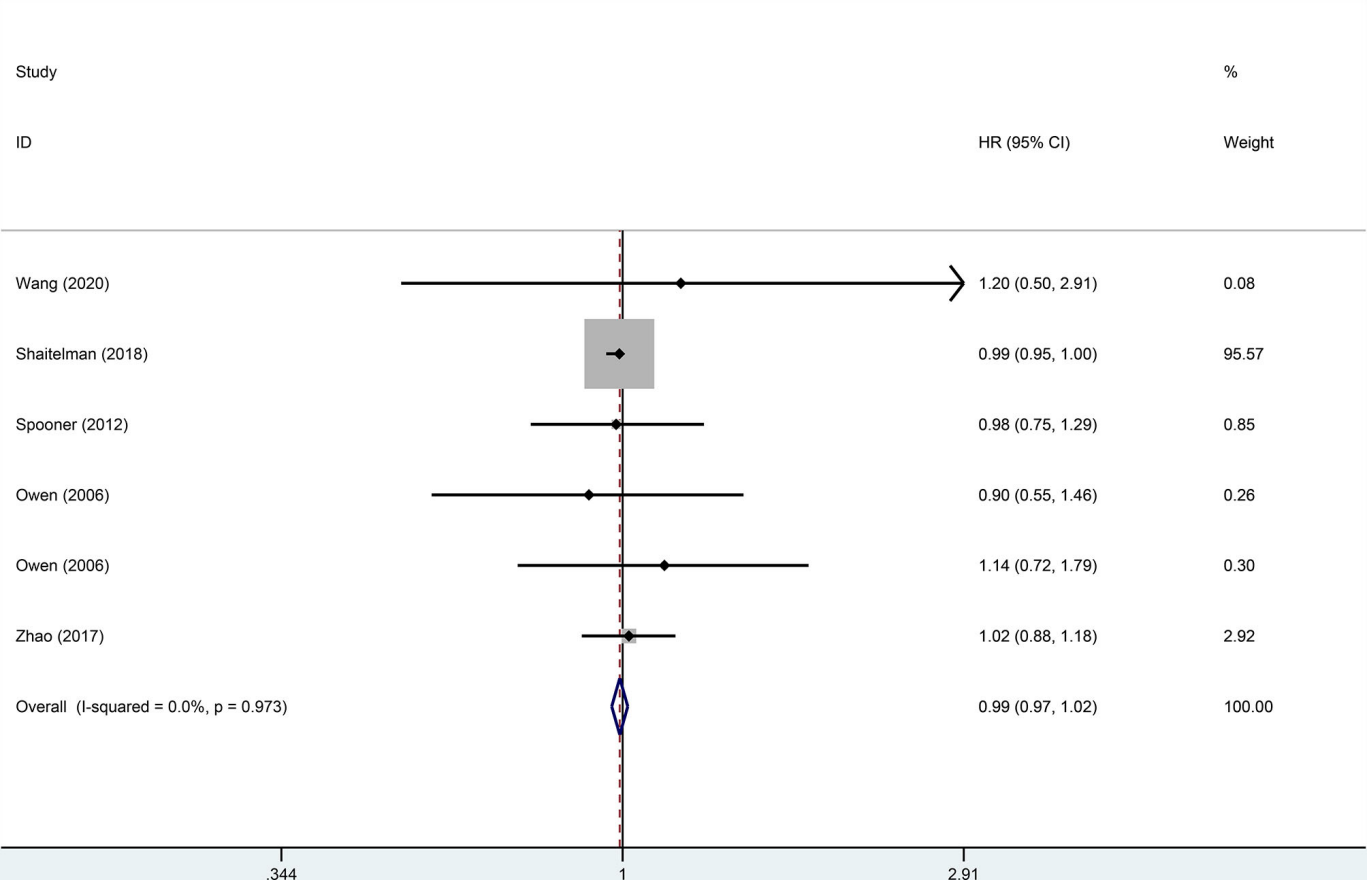
Forest plot of hypofractionated radiotherapy (HFRT) *vs*. conventional radiotherapy (CFRT) for relapse-free survival (*p* = 0.485).

**Figure 4:**
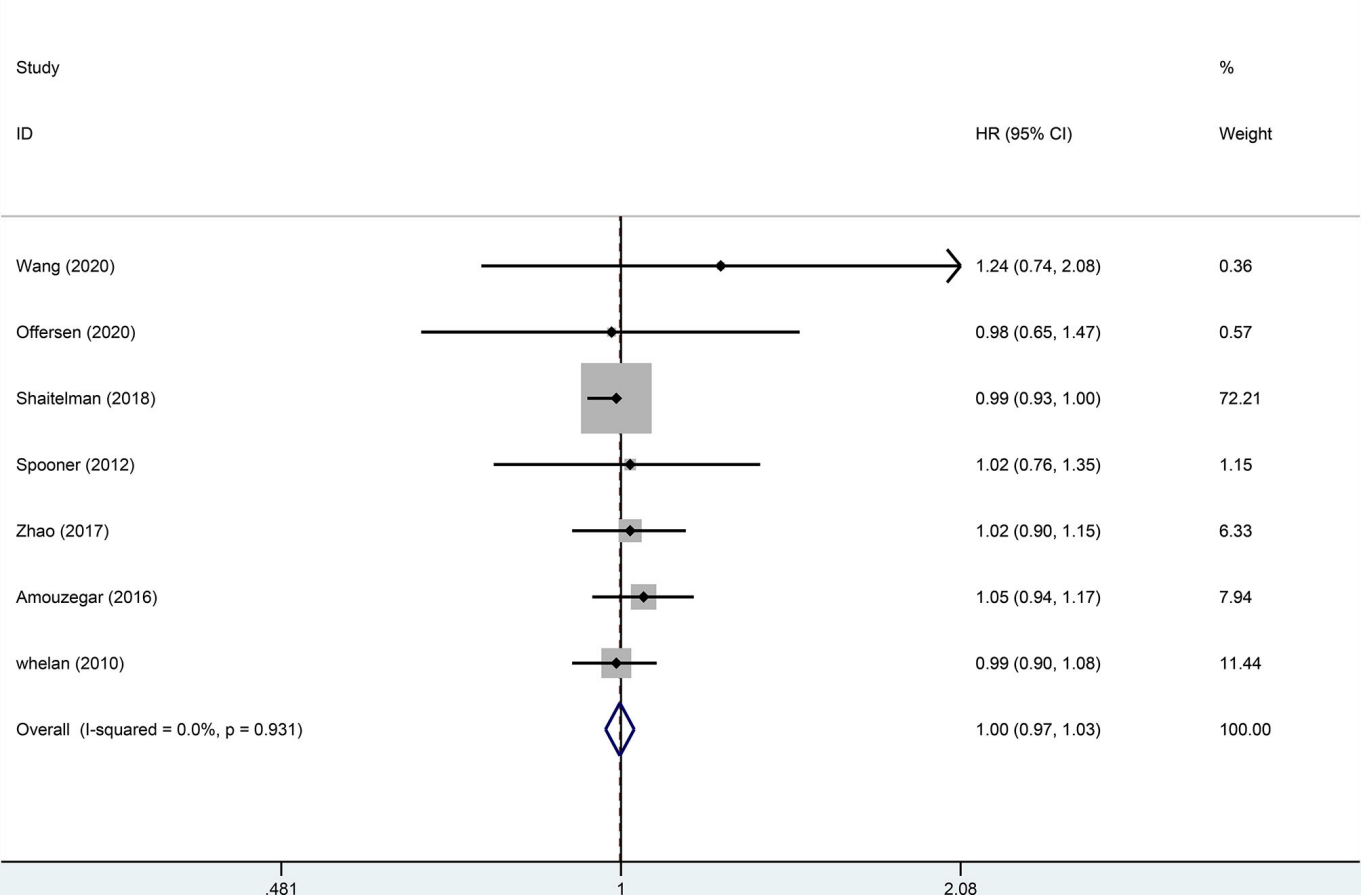
Forest plot of hypofractionated radiotherapy (HFRT) *vs*. conventional radiotherapy (CFRT) for overall survival (*p* = 0.879).

Four trials, with 1,499 patients in the experimental group and 1,039 in the control group, were included to evaluate local recurrence (LR). The results showed that there was no statistically significant difference in LR between the two groups (HR = 0.99, 95%CI = 0.97–1.02, *p* = 0.476). No heterogeneity was found in the included studies (*I* ^2^ = 0). A pooled analysis of 2,896 patients from five studies with low heterogeneity (*I* ^2^ = 0) indicated that the difference between HFRT and CFRT in relapse-free survival (RFS) was not statistically significant (HR = 0.99, 95%CI = 0.97–1.02, *p* = 0.485). The results of LR and RFS are shown in **Figures 2** and **3**. As for the analysis of OS, seven trials involving 2,309 patients receiving HFRT and 2,317 patients receiving CFRT were included for evaluation. It was discovered that there was no significant relationship between OS and radiotherapy dose (HR = 1.00, 95%CI = 0.97–1.03, *p* = 0.879) (**Figure 4**).

In addition to survival and recurrence, our primary concern was to assess the toxicity of HFRT. Compared with CFRT, less severe all grade acute skin toxicity (RR = 0.76, 95%CI = 0.61–0.94, *p* = 0.01) and breast atrophy (RR = 0.87, 95%CI = 0.80–0.95, *p* = 0.001), shorter induration (RR = 0.88, 95%CI = 0.79–0.97, *p* = 0.01), and fewer side effects in terms of pain (RR = 0.72, 95% CI = 0.51–1.01, *p* = 0.05) were observed for HFRT. The risk of pneumonitis (RR = 0.74, *p* = 0.08) was lower in patients receiving HFRT compared to those receiving CFRT, but the difference was not statistically significant. Additionally, the analysis results indicated that there was no statistical difference between HFRT and CFRT in many other adverse events, including all grade late skin toxicity (RR = 1.08, *p* = 0.55), dermatitis (RR = 0.79, *p* = 0.57), dyspigmentation (RR = 0.82, *p* = 0.15), edema (RR = 0.77, *p* = 0.10), telangiectasia (RR = 1.15, *p* = 0.35), and delayed toxic effects in subcutaneous tissues (RR = 0.95, *p* = 0.49). Detailed data are shown in **Table 2**.

**Table 2:** All grade adverse events for HFRT *vs*. CFRT.

HFRT *vs*. CFRT	No. of studies	Participants	RR	95%CI	*p*	Heterogeneity (*I* ^2^) (%)	Model
Acute skin toxicity	5	1,415	0.76	0.61–0.94	0.01	91	RE
Late skin toxicity	3	625	1.08	0.85–1.37	0.55	0	FE
Dermatitis	3	465	0.79	0.36–1.76	0.57	77	RE
Dyspigmentation	3	1,737	0.82	0.62–1.08	0.15	0	FE
Induration	6	7,002	0.88	0.79–0.97	0.01	0	FE
Edema	4	6,870	0.77	0.57–1.05	0.10	64	RE
Pneumonitis	2	789	0.74	0.52–1.04	0.08	0	FE
Pain	3	2,385	0.72	0.51–1.01	0.05	0	FE
Breast atrophy	2	4,690	0.87	0.80–0.95	0.001	0	FE
Telangiectasia	3	1,709	1.15	0.86–1.55	0.35	0	FE
Delayed toxic effects in subcutaneous tissues	2	545	0.95	0.81–1.11	0.49	0	FE

HFRT, hypofractionated radiotherapy; CFRT, conventional fractional radiotherapy; RR, risk ratio; CI, confidence interval.

With regard to moderate or marked adverse events, the results showed that HFRT can reduce the risk of acute skin toxicity (RR = 0.32, 95%CI = 0.15–0.69, *p* = 0.004) and breast atrophy (RR = 0.91, 95%CI = 0.84–0.98, *p* = 0.02) compared to CFRT. Also, there was no significant difference between modalities in moderate or marked late skin toxicity (RR = 0.95, *p* = 0.92), induration (RR = 0.93, *p* = 0.57), edema (RR = 0.81, *p* = 0.27), and delayed toxic effects in subcutaneous tissues (RR = 0.74, *p* = 0.54) after the two radiotherapies. Detailed data are shown in **Table 3**.

**Table 3:** Moderate or marked adverse events for HFRT *vs*. CFRT.

HFRT *vs*. CFRT	No. of studies	Participants	RR	95%CI	*p*	Heterogeneity (*I* ^2^) (%)	Model
Acute skin toxicity	3	1,195	0.32	0.15–0.69	0.004	27	FE
Late skin toxicity	2	545	0.95	0.34–2.67	0.92	0	FE
Induration	2	5,562	0.93	0.74–1.18	0.57	80	RE
Edema	2	5,562	0.81	0.56–1.18	0.27	81	RE
Breast atrophy	2	5,562	0.91	0.84–0.98	0.02	20	FE
Delayed toxic effects in subcutaneous tissues	2	545	0.74	0.28–1.95	0.54	0	FE

HFRT, hypofractionated radiotherapy; CFRT, conventional fractional radiotherapy; RR, risk ratio; CI, confidence interval; BC, breast cancer.

**Figure 5:**
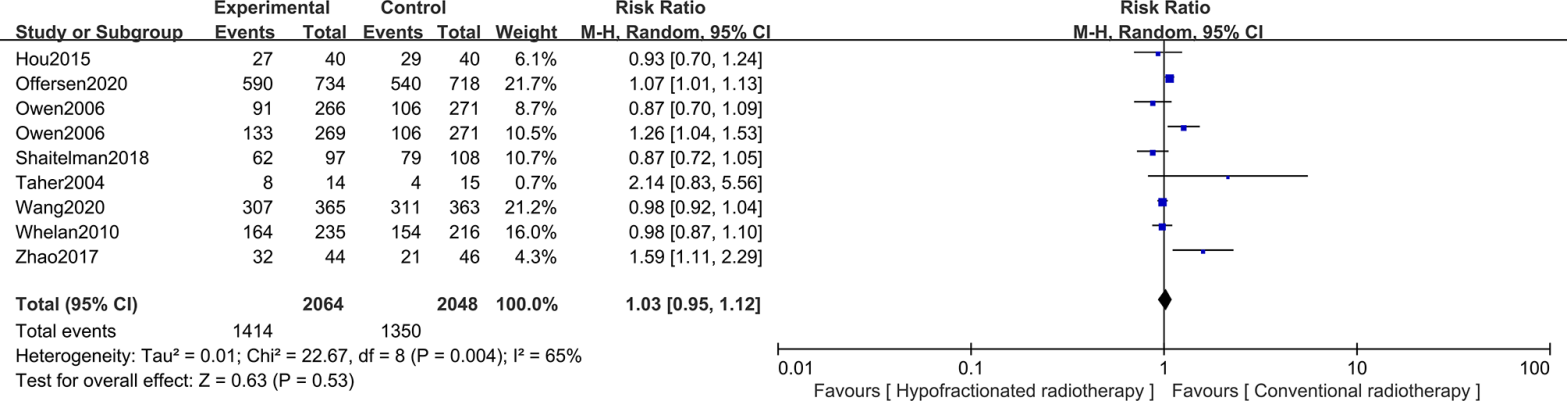
Forest plot of hypofractionated radiotherapy (HFRT) *vs*. conventional radiotherapy (CFRT) for cosmetic outcomes (*p* = 0.53).

For early breast cancer patients who had undergone breast-conserving surgery, whether the cosmetic intervention after radiotherapy is excellent/good or not is also worth considering. Hence, the cosmetic outcomes of surgery were analyzed based on 3,841 patients from eight studies, with 2,064 patients in the HFRT group and 1,777 patients in the CFRT group. Patients’ cosmetic outcomes were scored on a scale of “excellent,” “good,” “fair,” and “poor”. Pooled RR revealed no significant difference in excellent/good cosmetic outcomes between HFRT and CFRT (RR = 1.03, 95%CI = 0.95–1.12, *p* = 0.53). More detailed information is shown in **Figure 5**.

## Discussion

In 2018, the evidence-based guidelines provided by the American Society of Radiation Oncology (ASTRO) strongly recommend that, for women undergoing whole-breast irradiation (WBI) with invasive breast cancer, the preferred protocol is hypofractionated whole-breast irradiation (HF-WBI) at 15 fractions of 40 Gy or 16 fractions of 42.5 Gy (33). The guidelines further expanded the range of eligibility of patients to treat breast cancer with HF-WBI and suggest that neither age, tumor grade, nor chemotherapy is a contraindication for HFRT application. Several large clinical trials have laid the foundation for the HFRT plan. The results have shown that the data on local control, survival rate, and recurrence for HFRT are as effective as those for CFRT. Compared with CFRT, HFRT is also advantageous in terms of being relevant to fewer adverse events. Although HFRT has shown superiority to a certain extent, its use is still controversial for patients who suffer from ductal carcinoma *in situ* (DCIS) and with tumor bed boost, and there are also a few problems in cosmetic outcomes and onset of symptoms of adverse events. For example, it has been suggested that HFRT may increase the development of fibrosis in breast cancer patients (34). There are also studies suggesting that HFRT will enhance the brachial plexopathy rate (35). Therefore, a large amount of evidence is still necessary to consolidate the decision on the proper use of HFRT.

Our meta-analysis evaluated 14 RCTs and showed no statistically significant differences in LR, RFS, OS, cosmetic outcomes, or other adverse events between HFRT and CFRT. In addition, HFRT, compared to CFRT, treats the disease with relatively lower acute skin toxicity and breast atrophy, shorter induration, and less pain.

In terms of effectiveness, two previously published meta-analyses in the same topic analyzed LR and OS (36, 37). The results of our meta-analysis were consistent with their results, showing that there was no statistically significant difference between HFRT and CFRT in LR and OS. Additionally, HFRT and CFRT also showed similar results in RFS in our study. Different fractionated dose schemes mainly depend on the proliferative state of tumor tissue and the tolerance of normal tissue (38). In radiobiology, tissues are divided into early responding and late responding tissues according to the linear quadratic (LQ) model (39). The sensitivity of tissues to the radiotherapy fraction dose was quantified by the *α*/*β* value. Tissues with high *α*/*β* (>10 Gy) values are the early responding tissues with fast cell proliferation, and vice versa (40). Withers proposed, through the LQ model in radiobiology, that late responding tissues were more sensitive to changes in the fraction dose, which laid the foundation for the treatment with HFRT (41). Then, Douglas applied radiobiology theory to clinical review and concluded that HFRT is potentially effective and safe for tumor treatment (42). It is widely believed that the *α*/*β* value of tumor tissue is generally 8–10 Gy. CFRT is based on the assumption that breast cancer is less sensitive to changes in the fraction dose than is normal tissue; therefore, 2 Gy per fraction with a total dose of 50 Gy is capable of protecting healthy tissue from being damaged (43). However, according to the study by Yarnold et al., the *α*/*β* value of breast cancer was calculated and was inferred to be low, approximately 4 Gy, which falls in the range 0.75–5.01 Gy. Also, the *α*/*β* value of normal breast tissue is about 3 Gy, suggesting that the sensitivity of breast cancer tissue to dose segmentation was similar to that of normal tissue (44). In other words, HFRT could theoretically be similarly effective without a significant increase in adverse effects, making it more beneficial to breast cancer patients (45). HFRT in breast cancer is proposed as an improved approach against traditional radiotherapy based on the dynamics of breast cancer proliferation. The main purpose of HFRT is to protect normal tissues and specifically kill tumor cells with maximum lethality. Therefore, in this study, we used data to verify the authenticity of the theory.

When analyzing adverse events, we divided them into all grades and moderate/marked adverse events. Ninety-five percent of cancer patients treated with radiotherapy would more or less develop radiation dermatitis, including erythema, dry desquamation, and wet desquamation (46). Our analysis revealed that, compared to CFRT, HFRT has significantly lower all grade and moderate/marked acute skin toxicity. Zhou et al. (37) and Andrade et al. (36) came to the same conclusion that HFRT was associated with less grade 2/3 acute skin toxicity. Moreover, we also observed a significant improvement in terms of less pain suffered by patients in the HFRT group. The improvement in acute skin toxicity and pain relief may be due to the fact that acute toxicity is more dependent on the total dose than the fraction size (47). Thus, HFRT may minify the acute toxicity by decreasing the total dose. Breast induration is the possible outcome of advanced fibrosis following radiotherapy for breast cancer (48), and the possibility of breast fibrosis increased for 2 years after radiotherapy (49). It has been suggested that HFRT increases the incidence of fibrosis (34), but our data showed that the incidence of breast induration was improved in the HFRT group. The mechanism of HFRT developing less induration is not yet clear. One conjecture is that the reduction in the total dose led to this beneficial characteristic, or, from a radiobiological point of view, the limit to avoid chronic toxicity increase is 3.2–3.3 Gy per segment (50).

Our analysis showed that, compared to CFRT, fewer side effects in terms of all grade breast atrophy and moderate/marked breast atrophy were observed for HFRT. Breast atrophy in patients with breast cancer may be linked to the reduced estrogen levels. Some patients have received anti-estrogen-based endocrine therapy, thus losing estrogen stimulation and producing atrophy (51). However, it is still difficult to explain why HFRT is superior to CFRT in terms of breast atrophy, and no relevant studies have been reported to date.

The lung can become a threatening organ. This is because pneumonia is a common and a serious complication after radiotherapy for breast cancer (52). Severe radiation pneumonia has been reported to have a negative impact on the survival of breast cancer patients who had undergone radiotherapy (53). In this analysis, there was no statistically significant difference in pneumonia between the HFRT and CFRT groups. In fact, the incidence of radiation-induced lung injury was much lower under the modern treatment method (54). Meanwhile, pneumonia caused by chemotherapy drugs and radiation damage may accumulate (55). Therefore, the radiation-pneumonia with HFRT still requires further investigation.

Radiotherapy for breast cancer may involve radioactive exposure of the heart, which can cause ischemic heart disease (56). After radiotherapy for cancer in the left breast, it is easy to develop ischemic heart disease. This has been recognized as a rare but associated sequelae and is generally considered disadvantageous in terms of survival benefits when looking much further afield (57). Studies have suggested that there is no safe threshold to the heart for breast cancer radiotherapy. The damage is potentially threatening as long as there is a dose of radiotherapy. However, in the studies we included, the incidence of ischemic heart disease was relatively rare, and it was only in the Canadian trial that a few deaths were observed (15). In the study of Zhou et al., there was no significant statistical difference between HFRT and CFRT in the incidence of developing ischemic heart disease (37). The *α*/*β* ratio of the heart is relatively low. Studies have found that when the *α*/*β* is equal to 3 Gy, the bioequivalent dose of HFRT to the heart is lower than that of CFRT, which may be the reason why there was no statistical difference between HFRT and CFRT regarding ischemic heart disease (58). Unfortunately, due to the lack of data, our study was unable to provide data in this regard.

Andrade et al. (36) reported that HFRT had a better outcome than CFRT in other adverse events such as telangiectasia and breast edema, but we failed to come up with a similar conclusion. The different results in the analysis of telangiectasia may be due to the small sample size. We included 1,709 patients, while the meta-analysis of Andrade et al. included 5,167 patients. The inconsistent results regarding edema may be due to the inclusion of patients with partial mastectomy in their studies, which is not comparable to patients with breast-conserving surgery.

Cosmetic outcomes were evaluated in several studies, primarily by clinicians or nurses, based on the standard European Organization for Research and Treatment of Cancer/Radiation Therapy Oncology Group (EORTC/RTOG) cosmetic scoring system. Radiotherapy may lead to breast volume reduction, breast invagination, pigmented skin telangiectasia, subcutaneous tissue fibrosis, and other adverse reactions. It is undeniable that radiotherapy can result in negative impacts on the appearance of the breast (59). Studies have shown that patients’ dissatisfaction with the cosmetic outcomes may be associated with higher rates of depression (60); thus, we also focused our attention to cosmetic outcomes. Cosmetic outcomes may be related to age, tumor size, cancer stage, and breast volume systemic therapy, among others. In our analysis, excellent/good results in cosmetic outcomes were not significantly different between the HFRT and CFRT groups, which may be due to the improvement of skin toxicity and induration in patients.

Due to lack of data, we did not analyze the cost effectiveness of HFRT *versus* CFRT, but it has been proven by other studies that HFRT is more cost-effective than CFRT (61). One study has built models to estimate cost effectiveness from the perspective of the social and health sectors; the results revealed that HFRT is more cost-effective than CFRT for women with early breast cancer who need adjuvant radiotherapy (62). A study from the USA reported that HFRT could save the US healthcare system millions of dollars in healthcare costs if it follows evidence-based practice guidelines and if expert advice is chosen appropriately (63). In addition, despite population differences, recent studies have demonstrated for the first time that post-mastectomy HFRT is often more cost-effective than CFRT for women at high risk of breast cancer in China, France, and the United States (64).

In previous studies, the Canadian trial did not use a boost, the START trial used a boost, and Yarnold’s (14) study indicated that a boost after HFRT may increase the risk of late toxicity. The optimal fraction dose scheme for a boost is not yet clear. The recent BIG 3-07/TROG 07.01 trial is exploring this issue (65).

Our search was comprehensive, and in order to improve the reliability of the results with a higher level of evidence, the studies we included were all RCTs. Compared with previously published meta-analysis on the same topic, the study by Andrade et al. (36) had a limited sample size and included only six studies, while that by Zhou et al. (37) included non-RCT studies; the quality of evidence needs to be further confirmed. Moreover, some patients with mastectomy were included in their analyses. However, there are limitations in our study as well. Our subgroup analysis was inadequate due to the lack of data. In terms of the tumor stage of patients, our meta-analysis was not very rigorous. Although Wang’s study included patients with N2-3, we still included this study in our analysis. Some of the hotspot issues were not further stratified for analysis, such as whether a boost was used, DCIS patients, systemic treatment, and a stratified follow-up time.

From what has been discussed above, in patients with early breast cancer after breast-conserving surgery, HFRT and CFRT showed consistent outcomes in LR, RFS, and OS. HFRT is generally safe and does not differ from CFRT in terms of adverse events such as pneumonia, telangiectasia, and breast edema. Also, HFRT has better outcomes in acute skin toxicity, induration, breast atrophy, and pain. In addition, HFRT and CFRT have shown similar results regarding cosmetic outcomes. Currently, the safety and efficacy of HFRT have been examined to some extent, but it has not been fully utilized in clinical practice and needs to be further improved.

## Data Availability Statement

The original contributions presented in the study are included in the article/**Supplementary Material**. Further inquiries can be directed to the corresponding author.

## Author Contributions

LG, FW, and WD contributed to research conception and design. LG, RF, and KJ collected the data. JS, YS, HL, and MZ interpreted the data. All authors contributed to the drafting of the manuscript. All authors contributed to the article and approved the submitted version.

## Conflict of Interest

The authors declare that the research was conducted in the absence of any commercial or financial relationships that could be construed as a potential conflict of interest.

## Publisher’s Note

All claims expressed in this article are solely those of the authors and do not necessarily represent those of their affiliated organizations, or those of the publisher, the editors and the reviewers. Any product that may be evaluated in this article, or claim that may be made by its manufacturer, is not guaranteed or endorsed by the publisher.

## Abbreviations

RCT, randomized controlled trial; HR, hazard ratio; RR, risk ratio; CI, confidence interval; LR, local recurrence; RFS, relapse-free survival; OS, overall survival; CFRT, conventional radiotherapy; HFRT, hypofractionated radiotherapy; IARC, International Agency for Research on Cancer; EBCTCG, Early Breast Cancer Trialists’ Collaborative Group; PRISMA, Preferred Reporting Items for Systematic Reviews and Meta-Analyses; ASTRO, American Society of Radiation Oncology; WBI, whole-breast irradiation; HF-WBI, hypofractionated whole-breast irradiation; DCIS, ductal carcinoma *in situ*; LQ, linear quadratic; EORTC/RTOG, European Organization for Research and Treatment of Cancer/Radiation Therapy Oncology Group.
