# Supplementary material for: Comparing Hypofractionated With Conventional Fractionated Radiotherapy After Breast-Conserving Surgery for Early Breast Cancer: A Meta-Analysis of Randomized Controlled Trials
Source: Front Oncol. 2021 Oct 1;11:753209. doi: 10.3389/fonc.2021.753209 (PMC8518530; doi:10.3389/fonc.2021.753209)
Supplement: Supplementary file 1 [file Table_1.docx]

**Supplementary Table 1**. **Characteristics of included clinical trials in the meta-analysis.**

| Author | Year | Age range  (years) | Follow-up number | | Time period | Systemic therapy (Number) | | Outcome |
| --- | --- | --- | --- | --- | --- | --- | --- | --- |
|  |  |  | Experiment | Control |  | Chemotherapy | Hormonal therapy |  |
| Wang | 2020 | 18-70 | 365 | 364 | 2010-2018 | 119 | 238 | LR, DFS, OS, toxicity, cosmesis |
| Offersen | 2020 | 41-83 | 933 | 949 | NA | 456 | NA | LRR, OS, toxicity, cosmesis |
| Schmeel | 2020 | 19-84 | 70 | 70 | 2017-2019 | NA | NA | Toxicity, cosmesis |
| Shaitelman | 2018 | NA | 138 | 149 | 2011-2014 | 202 | NA | Toxicity, LRFS, LR, OS, patient report |
| Zhao | 2017 | NA | 53 | 54 | 2006-2016 | NA | NA | OS, DFS, LR, DFSR, toxicity, cosmesis |
| De Felice | 2017 | 39-82 | 58 | 62 | 2012-2015 | 25 | 102 | OS, DFS, FFS, toxicity |
| Amouzegar Hashem | 2016 | 37-80 | 30 | 22 | 2011-2016 | 14 | NA | Cosmetic, OS, LRR, LRFS |
| Hou | 2015 | NA | 40 | 40 | 2011-2013 | 27 | 34 | Local control, OS, toxicity, cosmesis |
| Fragkandrea | 2013 | 31-69 | 30 | 31 | NA | 16 | 56 | Pneumonitis |
| Haviland | 2013 | 25-85 | 750 | 749 | 1999-2004 | 245 | 1257 | LRR, DFS, OS, normal tissue effects, quality of life, toxicity |
|  |  |  | 737 |  |  |  |  |  |
|  |  | 23-86 | 1110 | 1105 |  | 155 | 1619 |  |
| Spooner | 2012 | 28-80 | 181 | 177 | NA | NA | NA | Relapses, OS, LRR, RFS |
| Whelan | 2010 | NA | 622 | 612 | 1993-2015 | 136 | 505 | LR, OS, toxicity, cosmesis |
| Owen | 2006 | NA | 466 | 470 | NA | 40 | 918 | Breast appearance, breast recurrence, toxicity |
|  |  |  | 474 |  |  |  |  |  |
| Taher | 2004 | 25-65 | 15 | 15 | NA | 17 | NA | Toxicity, cosmesis |

LR, local relapse; LRR, locoregional recurrence; OS, overall survival; DFS, disease free survival; DFSR, disease free survival rate; FFS, fibrosis free survival; RFS, Relapse-free survival; LRFS, locoregional relapse-free survival ; NA, not available; RCT, randomized controlled trial.
