# Supplementary material for: Comparing Hypofractionated With Conventional Fractionated Radiotherapy After Breast-Conserving Surgery for Early Breast Cancer: A Meta-Analysis of Randomized Controlled Trials
Source: Front Oncol. 2021 Oct 1;11:753209. doi: 10.3389/fonc.2021.753209 (PMC8518530; doi:10.3389/fonc.2021.753209)
Supplement: Supplementary file 2 [file Table_2.docx]

**Supplementary Table 2.** Quality assessment of studies included.

| Author, year,  Study (RCT) | Sequence  Generation | Allocation  Concealment | Blinding | Incomplete  outcome data | Selective  outcome reporting | Free of  other bias |
| --- | --- | --- | --- | --- | --- | --- |
| Wang, 2020 | low risk | low risk | high risk | low risk | low risk | unclear risk |
| Offersen, 2020 | low risk | low risk | high risk | low risk | unclear risk | unclear risk |
| Schmeel, 2020 | low risk | low risk | high risk | low risk | low risk | unclear risk |
| Shaitelman, 2018 | low risk | low risk | high risk | low risk | low risk | unclear risk |
| Zhao, 2017 | low risk | low risk | high risk | low risk | low risk | unclear risk |
| De Felice, 2017 | low risk | low risk | high risk | low risk | low risk | unclear risk |
| Amouzegar Hashemi, 2016 | low risk | low risk | high risk | high risk | low risk | unclear risk |
| Hou, 2015 | low risk | low risk | high risk | low risk | low risk | unclear risk |
| Fragkandrea, 2013 | low risk | low risk | high risk | low risk | high risk | unclear risk |
| Haviland, 2013 | low risk | low risk | high risk | low risk | low risk | unclear risk |
| Spooner, 2012 | low risk | low risk | high risk | low risk | low risk | unclear risk |
| Whelan, 2010 | low risk | low risk | high risk | low risk | low risk | unclear risk |
| Owen, 2006 | low risk | low risk | high risk | low risk | low risk | unclear risk |
| Taher, 2004 | low risk | low risk | high risk | unclear risk | low risk | unclear risk |

The RCTs were assessed by the Cochrane Collaboration’s tool. Risk of bias was assessed as “low risk”, “high risk” or “unclear risk”.
